# Fabrication and Assessment of Orodispersible Tablets Loaded with Cubosomes for the Improved Anticancer Activity of Simvastatin against the MDA-MB-231 Breast Cancer Cell Line

**DOI:** 10.3390/polym15071774

**Published:** 2023-04-02

**Authors:** Randa Mohammed Zaki, Amal El Sayeh Abou El Ela, Alanood S. Almurshedi, Basmah Nasser Aldosari, Abdullah A. Aldossari, Mohamed A. Ibrahim

**Affiliations:** 1 Department of Pharmaceutics, College of Pharmacy, Prince Sattam Bin Abdulaziz University, Al-Kharj 11942, Saudi Arabia; 2 Department of Pharmaceutics and Industrial Pharmacy, Faculty of Pharmacy, Beni-Suef University, Beni-Suef 62514, Egypt; 3 Department of Pharmaceutics, College of Pharmacy, King Saud University, Riyadh 11451, Saudi Arabia; 4 Department of Pharmacology and Toxicology, College of Pharmacy, King Saud University, Riyadh 11451, Saudi Arabia

**Keywords:** cubosomes, simvastatin, breast cancer

## Abstract

Various factors limit the use of simvastatin as an anticancer drug. Therefore, this study aimed to analyse simvastatin (SIM)-loaded cubosome efficacy against breast cancer. SIM-loaded cubosomes were prepared using the emulsification method using different glyceryl monooleate, Pluronic F127 (PF-127), and polyvinyl alcohol (PVA) ratios. The best cubosomal formula was subjected to an in vitro cytotoxicity analysis using the human breast cancer cell line, MDA-MB-231 (MDA) (ATCC, HTB-26), and formulated as oral disintegrating tablets through direct compression. PF-127 and PVA positively affected drug loading, and the entrapment efficiency percentage of different SIM-cubosomal formulations ranged from 33.52% to 80.80%. Vesicle size ranged from 181.9 ± 0.50 to 316.6 ± 1.25 nm. PF-127 enhanced in vitro SIM release from cubosome formulations due to its solubilising action on SIM. The in vitro dissolution analysis indicated that SIM exhibited an initial dissolution of 10.4 ± 0.25% within the first 5 min, and 63.5 ± 0.29% of the loaded drug was released after 1 h. Moreover, cubosome formula F3 at 25 and 50 µg/mL doses significantly decreased MDA cell viability compared to the 12.5 µg/mL dose. The untreated SIM suspension and drug-free cubosomes at all doses had no significant influence on MDA cell viability compared to the control.

## 1. Introduction

Simvastatin (SIM), a lipophilic statin, is a commonly prescribed cholesterol-lowering drug accepted by the Food and Drug Administration (FDA) to treat high cholesterol levels related to high low-density lipoproteins (LDL) cholesterol levels and heart disease [1,2]. The cholesterol-lowering effect of SIM is due to the inhibition of the 3-hydroxy-3-methyl-glutaryl-coenzyme A (HMG-CoA) reductase enzyme used for cholesterol synthesis [3]. SIM decreases LDL cholesterol by 50% and increases high-density lipoprotein (HDL) cholesterol by 5–10% [4]. In addition to its lipid-lowering effect, SIM has a direct anti-atherosclerotic effect on arterial walls, significantly inhibiting heart disease [5]. Moreover, statins have pleiotropic effects on neurological diseases, inflammation, diabetes, and cancer [6]. Considering their anticancer, cost-effective, and less toxic effects compared to conventional chemotherapeutic agents, statins have attracted significant attention as anticancer drugs in recent decades [7]. Many studies have demonstrated the beneficial effects of statins against cancer [8,9,10,11,12]. However, SIM has several limitations. Owing to its insufficient solubility, it is poorly and incompletely absorbed from the gastrointestinal tract and subject to first-pass metabolism, resulting in low bioavailability [13]. Moreover, SIM has a short biological half-life (1.5–2 h) [14]. These factors lower the effectiveness of SIM.

Therefore, incorporating SIM into lipid-based nanocarriers, such as cubosomes, could overcome these limitations. Cubosomes can enhance the delivery of poorly aqueous-soluble drugs by improving their solubility [15,16,17].

Cubosomes are innovative self-assembled nanodispersions with bicontinuous cubic phase liquid crystals stabilised by surfactants [18]. They are prepared by emulsifying lipid phases, mainly glyceryl monooleate (GMOs), in water [19,20]. Cubosomes are a common drug delivery system owing to their various properties. They can encapsulate hydrophilic, lipophilic, and amphiphilic drugs [21]. Additionally, their benefits include simple manufacturing methods, composition from biodegradable materials, targeted or sustained release of drugs, and the ability to hold larger drug quantities because of their liquid crystalline structure [22]. They are more stable, less viscous and have a less hydrophobic core than liposomes [23,24].

Swarnakar et al. [25] reported that cubosomes enhanced oral delivery and anticancer activity due to the improvement in the circulation half-life and accumulation in the tumour, resulting from enhanced permeation and retention (EPR) effect. Furthermore, their lipid nature enables them to target the lymphatic system [26]. Many drugs can be administered orally via the lymphatic system to avoid hepatic first-pass metabolism [27]. Nanoparticles that contain lipid increase drug uptake into the lymphatic circulation, possibly because of their lipid content and small size [28]. Furthermore, various chemotherapeutic drugs have been introduced into cubosomes to enhance their accumulation and efficacy against cancer cells [29,30,31,32,33,34].

This study aimed to evaluate the efficacy of SIM-loaded cubosomes against breast cancer. SIM-loaded cubosomes were prepared using emulsification method using different glyceryl monooleate, Pluronic F127 (PF-127), and polyvinyl alcohol (PVA) ratios. SIM-loaded cubosomal formulations were characterised for different parameters; entrapment efficiency percentage (EE%), vesicles’ sizes, zeta potential, vesicle morphology by transmission electron microscope (TEM) and in vitro drug release. The best cubosomal formula was subjected to an in vitro cytotoxicity analysis using the human breast cancer cell line, MDA-MB-231 (MDA) (ATCC, HTB-26), and formulated as oral disintegrating tablets through direct compression.

## 2. Materials and Methods

### 2.1. Materials

SIM was kindly provided by Al Jazeera Pharmaceutical Company (Riyadh, Saudi Arabia). GMO, Pluronic F127 (PF-127), and polyvinylpyrrolidone (PVP) were purchased from Sigma-Aldrich (Saint Louis, MO, USA). Spray-dried mannitol, mannogem^TM^ EZ, was supplied by SPI (Grand Haven, NY, USA). Spray-dried lactose monohydrate (FlowLac ^®^100) and crospovidone (CPV) were kindly provided by Meggle (Wasserburg, Germany) and Riyadh Pharma (Riyadh, Saudi Arabia), respectively. Microcrystalline cellulose (Avicel PH101) was purchased from Serva Feinbiochemica (Heidelberg, Germany). Magnesium stearate was obtained from Riedel-de Haën (Seelze, Germany).

### 2.2. Methods

#### 2.2.1. Preparation of SIM-Loaded Cubosomes

The SIM-loaded cubosomes were prepared using emulsification [35]. Different ratios of GMO (lipid phase) and PF-127 (surfactant) were used (Table 1) such that the concentration of GMO/PF-127 mixture was 5% *w*/*w* for the total weight of the cubosomal dispersion. PVA was used as a stabiliser for the cubosomal dispersion and was added at concentrations of 0%, 2.5%, and 5% *w*/*w* for the dispersed phase. Briefly, GMO and PF-127 were melted on a hot plate at 70 °C, and SIM was added to the melted mixture. PVA was dissolved in 10 mL water at 70 °C, and the melted mixture was added dropwise to the aqueous phase at the same temperature under mechanical stirring at 1500 rpm for 2 h. Finally, the formed cubosomal dispersions were allowed to cool to room temperature and were ultrasonicated for 10 min to reduce their size to the nanoscale [36].

#### 2.2.2. Characterisation of SIM-Loaded Cubosomes

- Determination of Entrapment Efficiency percentage (EE%)

Different cubosomal formulations were centrifuged using a cooling centrifuge (SIGMA 3–30 K, Sigma, Steinheim, Germany) at 16,000 rpm for 1 h at 4 °C to separate the cubosomal nanovesicles from the free un-entrapped SIM. The supernatant containing the free SIM was filtered, properly diluted, and quantified for SIM concentration using an ultraviolet (UV) spectrophotometer (Shimadzu UV-1800, Kyoto, Japan) at a predetermined λmax (238 nm). The EE% was computed using the following equation: [20,37,38]

EE% = (TD − FD)/TD × 100(1)

where EE% is the entrapment efficiency percentage, TD is the total amount of drug, and FD is the free amount of drug in the supernatant.

- Vesicle Size Distribution and Zeta Potential analysis.

Particle size and zeta potential are common constraints that can influence the performance of SIM-loaded cubosomes. In this study, PF-127 and PVA were used as stabilisers during cubosome preparation. The polydispersity index (PDI) and vesicle size of the SIM-loaded cubosome formulations were analysed using dynamic light scattering using a Zetasizer Nano ZS (Malvern Instruments, Ltd., Worcestershire, UK). Briefly, 1 mL of cubosomal formulation was diluted to 100 mL with deionised water and vortexed for 2 min then placed in the clean cuvette of the apparatus and equilibrated at 25 °C. Zeta potential was assessed using the Zetasizer Nano ZS (Malvern Instruments, Ltd., Worcestershire, UK) based on electrophoretic movement after appropriate dilution. The measurements were repeated three times and the data are offered as average values.

- In Vitro Release

The measurement of the release of SIM from different cubosomal formulations compared to the SIM suspension was conducted using the dialysis bag method [39]. An accurate quantity of each formulation, equivalent to 20 mg SIM, was placed in the dialysis bags, which were suspended in a dissolution medium (250 mL phosphate buffer, pH [6.8]) [39]. The dissolution apparatus (Pharm Test, Hainburg, Germany) was set at 37 °C and stirred at a speed of 100 rpm. Samples (5 mL) were obtained at different time points (0.5, 1, 1.5, 2, 2.5, 3, 3.5, and 4 h) and immediately exchanged with an identical volume of a fresh dissolution medium. The quantity of SIM in different samples was assessed spectrophotometrically at 238 nm. The measurements were repeated three times and the data are offered as average values. the percentage of SIM released was computed as follows [40]:

(2) $$\mathrm{Qn}=\frac{\mathrm{Cn}\times \mathrm{Vr}+\sum_{i=1}^{n-1}\mathrm{Ci}\times \mathrm{Vs}}{\mathrm{initial}\;\mathrm{drug}\;\mathrm{content}}$$

where:

Qn: cumulative percentage of SIM released
Cn: concentration of SIM in the dissolution medium at the nth sample
Vr: volume of dissolution medium
Vs: volume of sample
$\overset{n-1}{\underset{i=1}{\sum }}\mathrm{Ci}\overset{n-1}{\underset{i=1}{\sum }}\mathrm{Ci}$: the summation of the concentrations measured formerly

The release profiles of different cubosomal formulations compared to the SIM suspension were determined by plotting the cumulative percentage of SIM released (Qn) at different time intervals against time. To determine the mechanism of drug release, the data were fitted into various kinetic models, including zero-order, first-order, and Higuchi diffusion models.

#### 2.2.3. Characterisation of the Selected SIM-Loaded Cubosomal Formula

- X-ray Diffraction Analysis

X-ray diffraction (XRD) patterns of SIM, PF-127, mannitol, and SIM-loaded cubosomes were obtained by a RIGAKU diffractometer (Kyoto, Japan) fitted with a curved graphite crystal monochromator (Kyoto, Japan), programmed deflection split, and programmed controller PW/1710. Cu K α radiation was the target, working at 40 KV and 40 mA (Cu K α = 1.5418 Å). The patterns of different samples were obtained by an uninterrupted examination manner with 2Ɵ° ranged from 4° to 60°.

- Fourier-Transform Infrared Spectroscopy
- The Fourier-Transform Infrared Spectroscopy (FTIR) spectra of SIM, PF-127, mannitol and SIM-loaded cubosome were obtained by an FTIR Perkin Elmer spectrophotometer (Spectrum BX, USA). Firstly, an accurate weight of each sample was mixed with potassium bromide (spectroscopic grade) then compressed into disks with a hydraulic press. The scan was performed from 4000 to 600 cm^−1^. The results were investigated by Perkin Elmer software (Spectrum V5.3.1, Milford, MA, USA).
- Transmission Electron Microscopy (TEM)
- To assess the morphological characteristics of the selected formula, a transmission electron microscope (TEM; JEOL JEM-1010, Tokyo, Japan) was utilized. Firstly, the sample was sonicated for 1 min and suitably diluted then one drop from the formula was put on a carbon-coated copper grid. After that, phosphotungstic acid was added to stain the sample. Finally, the sample was allowed to be dried in the air for 5 min and image was taken at a magnification power of 10,000×. The apparatus was operated at 80 KV.

#### 2.2.4. Manufacture of Directly Compressed Oral Disintegrating Tablets Containing the Selected Cubosomal Formula

Oral disintegrating tablets (ODTs) containing SIM-cubosome formula (F3), which showed the highest drug release rate of 81.71 ± 0.445% after 4 h, were manufactured through direct compression. First, to convert the liquid cubosome into a powder before mixing with tablet excipients and tablet compression, the selected cubosomal formula was freeze-dried with a formula weight of mannitol (116 mg) at −60 °C at a vacuum pressure of ˂1 Mbar (Alpha 1–4 LD Plus, Martin Christ Gefriertrocknung Anlagen GmbH, Osterode am Harz, Germany). Tablet ingredients of tablet weight were mixed as follows: spray-dried cubosome formula with mannitol was 239.5 (equivalent to 10 mg SIM); magnesium stearate was 4 mg; CPV was 40 mg and Avicel PH 101 was to 400 mg). The corresponding amounts of the SIM-cubosome formula (F3) freeze-dried with mannitol, equivalent to 10 mg SIM, were mixed with the formula weight of Avicel pH 101 for 5 min using a Turbula mixer (Erweka, S2Y, Heusenstamm, Germany). Next, the formula weight of tablets’ superdisintegrant (CPV; 10% of tablet weight) was added, and mixing was continued for 5 min. Finally, the formula weighted amount of the lubricant (magnesium stearate; resembling 1% tablet weight) was mixed with the other powder tablets’ excipients for a further 2 min using the Turbula mixer. The blended powder of tablets’ excipients was compressed into shallow concave tablets weighing 400 mg applying 9 mm flat punches in a Korsh single-punch tablet press (Siemens, Munich, Germany).

#### 2.2.5. Characterization of SIM Cubosomes-Loaded ODTs

- Weight Variation

Tablets weight was evaluated by weighing 20 tablets individually using analytical balance (Shimadzu, EB-3200D, Kyoto, Japan). The average tablet weight as well as standard deviation were computed.

- Tablets’ Hardness

Pharma test hardness tester (GmbH, Hainburg, Germany) was used to measure the hardness of 10 tablets of defined thickness and weight. The average tablet hardness as well as standard deviation were then recorded.

- Friability

The friability of ODTs containing SIM or the untreated drug was calculated in accordance to the guidelines of USP 30-NF25. Briefly, 10 tablets were weighed (W1) and sited in a friabilator (Erweka, TA3R, Heusenstamm, Germany) then permitted to revolve at 25 rpm for 4 min. Thereafter, the fines were removed (W2) and the tablets were reweighed. The percentage loss was computed as follows:

100 × (W1 − W2)/W1(3)

- In Vitro Tablet Disintegration

The in vitro disintegration of the manufactured ODTS containing SIM cubosomes was evaluated in accordance to the USP 30-NF25 guidelines using a disintegration tester (Electrolab ED-21, Mumbai, India). One tablet was located in each of the six tubes of the disintegration tester basket. The baskets contained an immersion fluid which is phosphate buffer (pH 6.8), and the temperature was maintained at 37 ± 0.5 °C. The apparatus was then operated, and the time required for the complete disintegration of each tablet and standard deviation, as well as relative standard deviation were computed.

- In Vitro Dissolution Analysis

The in vitro dissolution profiles of pure SIM and SIM nanoparticles from the manufactured ODTs formulations were determined using USP II dissolution apparatus (Logan instruments Corp., Milford, MA, USA) according to the USP 40-NF35 SIM monograph. The dissolution experiment was conducted in triplicate using 500 mL of phosphate buffer as a dissolution medium (pH 6.8 ± 0.05) at 37 ± 0.5 °C and kept stirred at 50 rpm. Samples (5 mL) were withdrawn at predetermined time intervals (5, 10, 15, 30, 45 and 60 min) using a millipore filter (0.22 µM) and diluted appropriately. The absorbance was assessed spectrophotometrically at 238 nm compared to an appropriate blank.

#### 2.2.6. In Vitro Cytotoxicity Analysis

- Cell Culture

The human breast cancer cell line, MDA MB-231 (MDA) (ATCC, HTB-26), was cultured at a density of ~3 × 10^5^ cells/mL in a basal medium containing 1:1 Dulbecco’s modified Eagle’s medium 1× (DMEM 1×), 10% foetal bovine serum (FBS), and 1% streptomycin/penicillin (100 μg/mL and 100 units/mL, respectively). Cells were maintained in a humidified incubator containing 5% CO_2_ at 37 °C until they reached confluence and were passaged by trypsinisation (300 μL) in T-75 flasks. Cells from passages 3 to 10 were used for all experiments. All treatments were performed in a full culture medium containing FBS and streptomycin/penicillin.

- MTT Assay

The 3-(4,5-dimethylthiazolyl-2)-2,5-diphenyltetrazolium bromide (MTT) assay is a colourimetric method used to estimate cell proliferation and survival. This assay was used to evaluate the cytotoxicity of SIM, loaded cubosomes, and unloaded cubosomes on MDA cells. The cells were cultured as described in this section. The cells were then harvested and trypsinised for counting. Next, 1 × 10^4^ cells/well was seeded in 96-well plates. The plates were incubated for 24 h. In the survival experiments, viability was estimated in cells incubated with SIM, loaded cubosomes, or unloaded cubosomes (12.5, 25, and 50 µg/mL) for 24 h. Once the cells were incubated under the assigned conditions, 10 μL of MTT (5 mg/mL phosphate buffered saline [PBS]) reagent was added to each well for 30 min until the purple precipitate was viable. Next, 100 μL of detergent reagent (dimethyl sulfoxide [DMSO]) was added at room temperature while shaken for 5 min. Finally, absorbance at 570 nm was recorded using a microplate reader (the level of formazan formation is directly proportional to cell viability).

## 3. Results and Discussion

### 3.1. Entrapment Efficiency Percentage (EE%)

The EE% of different SIM-loaded cubosomes ranged from 33.52% to 80.80% (Table 1), indicating the effective entrapment of SIM into the cubosomes, making cubosomes a successful delivery system for SIM.

The positive effect of PF-127 and PVA on EE% may be attributed to their use as stabilisers to stabilise the cubosomal dispersion by forming a layer over the cubosomal nanovesicles; this layer could have retained an excessive amount of SIM, consequently enhancing the EE%. Additionally, the high attraction between the lipophilic drug, SIM, and the lipophilic part of the cubosomal bilayer facilitates high SIM encapsulation in the cubosomes [41]. For this reason, F6 and F9 showed the highest entrapment efficiency as they contain the highest percentage of PF 127 (10%) along with PVA. Moreover, F9 showed higher entrapment efficiency than F6 due to the higher PVA content in F9. The values of EE% were arranged as follows: F9 > F6 > F8 > F3 > F5 > F2 > F7 > F4 > F1.

### 3.2. Vesicle Size Distribution and Zeta Potential Analysis

All cubosomes analysed were in the nanometre range (Table 1). The average vesicle sizes ranged from 181.9 ± 0.50 to 316.6 ± 1.25 nm for F9 and F3, respectively. PF-127 stabiliser concentration affected the average vesicle size of cubosomes (Table 1). Increasing PF-127 concentration from 2.5% to 5% significantly increased the average vesicle sizes of the cubosomes (*p*-value < 0.05), as shown in the F1 to F2, respectively. Further increment to 10% resulted in a non-significant increase in the vesicle size. The vesicle size of the formulated cubosomes increased in the presence of a surfactant, possibly due to the reduction in the GMO (lipid phase) concentration in these fabricated formulations.

However, increasing the PVA stabiliser concentration from 0% to 2.5% significantly decreased average vesicle size (*p*-value < 0.05). Further increment to 5% slightly decreased the particle size (Table 1). This result is consistent with that of Esposito et al. [35], which indicated that the highest concentrations of PF-127 (10%, F9) and PVA (5%, F9) could influence the steric stability of the system and disrupt the crystalline structure of the dispersion. These results suggest that the combined effects of PF-127 in the presence of different concentrations of PVA could have an additional impact on vesicle size.

PDI points to the homogeneity of particles, and it has values ranged from 0.0 to 1.0. As the value approaches zero, the particles homogeneity increases [42,43]. SIM-loaded cubosomes had PDI values ranged from 0.292 ± 0.04 to 0.677 ± 0.02 (Table 1), indicating a moderately homogenous particle distribution and size.

The zeta potential values indicate the electrical charge on the surface of the vesicles, which is a mostly important parameter that influences the behaviour of cubosomes. A high zeta potential value indicates a high stable system because of the strong repulsion between the electrical charges on the surface of the vesicles that prevents nanovesicle agglomeration [44]. The obtained SIM-loaded cubosomes had zeta potential values in negative sign ranged from −46.0 ± 0.61 to −57.9 ± 0.11 for F3 and F4, respectively. The extent of this value was adequately great, providing good stability to the vesicles (Table 1).

### 3.3. In Vitro Release

**Figure 1:**
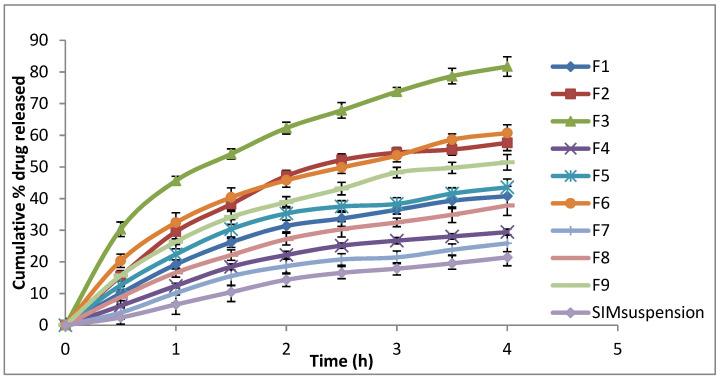
In vitro release profile of different SIM-loaded cubosomes.

The release profiles of the different cubosomal formulations compared to the SIM suspension are shown in Figure 1. All cubosomal formulations showed improved SIM release compared to the SIM suspension. The cumulative percentage release of SIM after 4 h from cubosomal formulations ranged from 25.88% to 81.71% compared to the 21.43% SIM release from the SIM suspension.

PVA is a polyol known as a brilliant size-controlling agent and stabiliser [41]. PVA negatively affected SIM release. The formulations without PVA had a higher percentage release than those containing PVA, corresponding to the same percentage of PF-127, consistent with the finding of Hadel et al. [41]. Moreover, PF-127 positively affected SIM release. PF-127 concentration increase amplified the percentage release of SIM, possibly because of the ability of PF-127 to solubilise SIM in the dissolution medium, facilitating SIM release from cubosome nanovesicles. These results are consistent with those of Salah et al. [45] and Omar et al. [46].

Kinetic release data are presented in Table 2. A linear relationship was observed between SIM release and R2. Moreover, the highest values of R2 were observed with Higuchi diffusion, confirming that the release followed the Higuchi diffusion model [47].

### 3.4. Characterisation of the Selected SIM-Loaded Cubosomal Formula

#### 3.4.1. X-ray Diffraction Analysis

**Figure 2:**
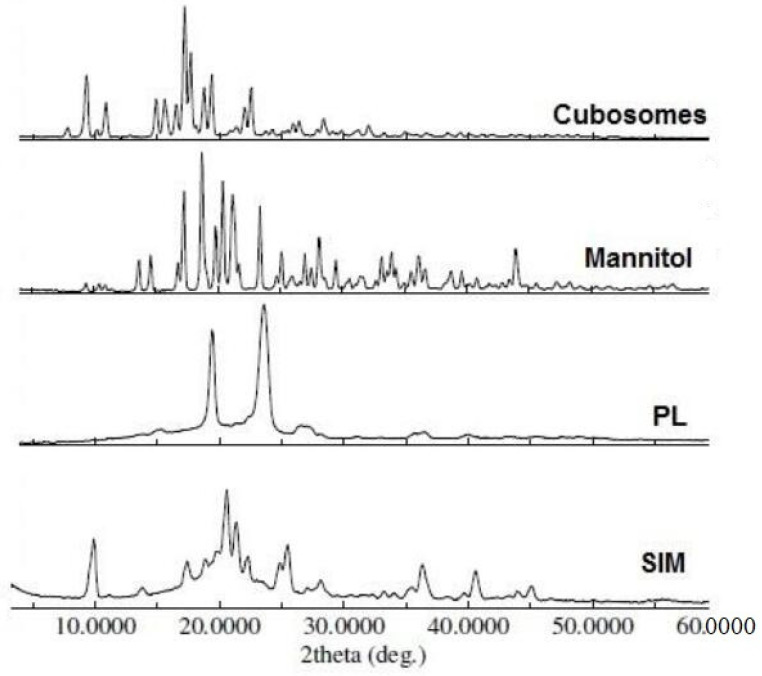
XRPD spectra of SIM, PF-127, mannitol, and SIM-loaded cubosomes.

XRD analysis was performed to assess the effect of the cubosome formula on the crystallinity of SIM compared to that of untreated SIM. The characteristic XRD spectra of untreated SIM, PF-127, mannitol, and cubosome formulations are illustrated in Figure 2.

The XRD pattern of the untreated SIM showed numerous distinctive peaks in the region of 7° to 60° 2θ of 9.9°, 20.6°, 21.3°, and 25.5°, indicating the crystalline nature of SIM. PF-127 exhibited two diffraction peaks at 19.4 and 23.6 2θ. Moreover, mannitol exhibited diffraction peaks at 17.3, 18.7, 20.4 and 21.4 2θ.

Figure 2 shows the XRD of the SIM-cubosome formula. No crystalline change was observed in the formula compared to the untreated SIM. The diffraction peaks observed in the spectra were due to the overlapping diffraction peaks of the other excipients and SIM.

#### 3.4.2. FTIR Analysis

**Figure 3:**
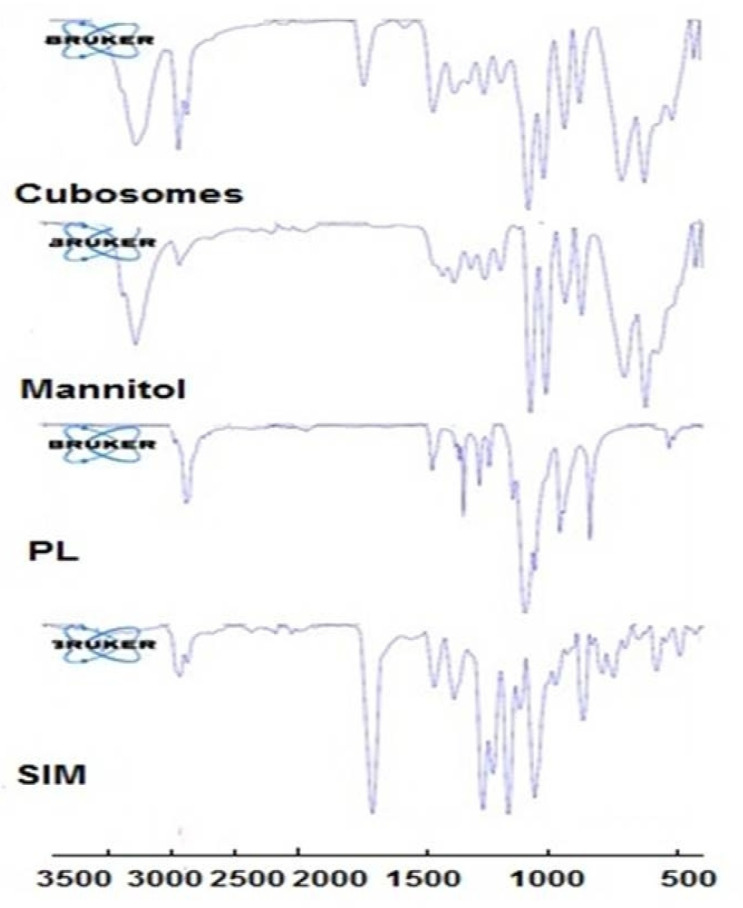
FTIR spectra of SIM, PF-127, mannitol and SIM-loaded cubosome.

Figure 3 shows the FTIR spectra of the untreated SIM. Primary determinant peaks were observed around 3546 cm^−1^ (-OH stretching), 2957 cm^−1^ (-CH stretching), 1703 cm^−1^ (C=O stretching), and 1164 cm^−1^ (-C-O stretching). Mannitol showed a broad band at 3280 cm^−1^ (-OH stretching vibration), while PF-127 showed a sharp band at 2878 cm^−1^ (-OH stretching vibration and hydrogen bonding). The OH stretching band of the drug at 3546 cm^−1^ disappeared completely. In addition, the C=O stretching at 1703 cm^−1^ shifted to 1730 cm^−1^ (˃27 cm^−1^). Moreover, the CH stretching band of Pluronic F 127 was shifted from 1466 to 141,454 cm^−1^. These results suggest an interaction between SIM and cubosome ingredients in the form of hydrogen bonding, which might participate in enhancing drug dissolution [48].

#### 3.4.3. Transmission Electron Microscopy (TEM)

**Figure 4:**
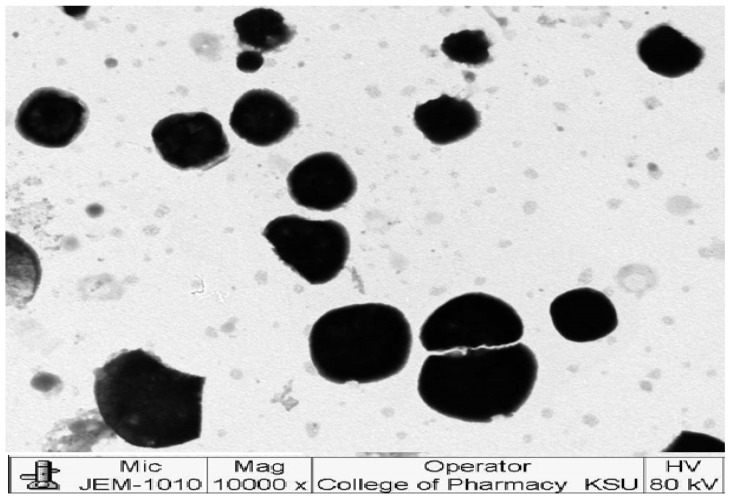
TEM image of the selected formula.

As shown in Figure 4, the TEM image of the selected cubosomal formula revealed well-dispersed cubosomes with irregular shapes. No aggregates were found, indicating the high stability of the cubosomes and this observation ratifies the results of the Zeta potential values.

### 3.5. Evaluation of ODTs Containing SIM Cubosomes

The established ODTs, containing a cubosomal formula equivalent to 10 mg of SIM, were effectively fabricated using the direct compression method. The ODTs showed an acceptable friability (˂1%), an average hardness of 2.1 ± 0.18 kp, and an average weight of 389.7 ± 7.8 mg with a drug content of 9.54 ± 0.62 mg. The prepared tablets showed rapid disintegration; the disintegration time for all six tablets was 38.4 ± 1.8 s.

**Figure 5:**
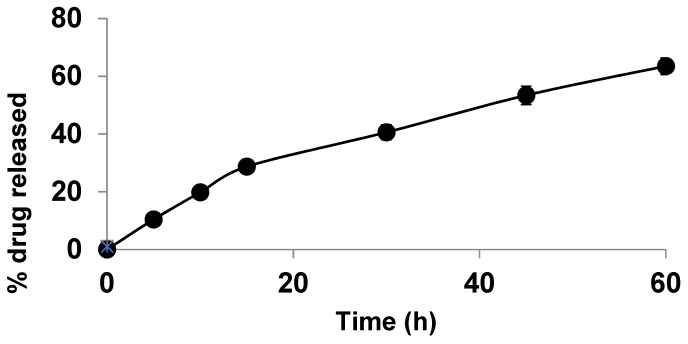
In vitro dissolution of SIM from ODTs containing drug-loaded cubosomal formula F3.

Figure 5 shows the in vitro dissolution pattern of SIM from ODTs containing the cubosomal formula F3. The drug showed an initial dissolution of 10.4 ± 0.25% within the first 5 min, and 63.5 ± 0.29% of the loaded drug was released within 1 h. The drug was slightly enhanced in dissolution from the tablets compared to the liquid cubosomal formula, possibly because of the effect of the hydrophilic additive (mannitol) and the rapid disintegration of the tablets due to the effect of the superdisintegrant.

### 3.6. In Vitro Cytotoxicity Analysis

**Figure 6:**
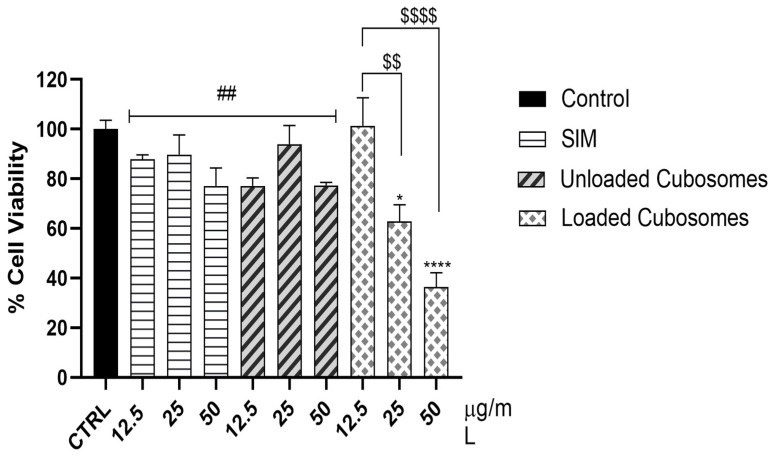
Cell viability of human breast cancer cell line, MDA, after incubation with SIM, unloaded cubosomes and loaded cubosomes for 24 h. Values are expressed as mean ± standard error of the mean (SEM) (n = 3/group). * Significant difference from control (*p* < 0.05). **** Significant difference from control (*p* < 0.0001). ## Significant difference from 50 µg/mL (*p* < 0.01). $$ Significant difference from 25 µg/mL (*p* < 0.01). $$$$ Significant difference from 50 µg/mL (*p* < 0.0001).

The MTT results revealed that SIM-loaded cubosomes significantly dose-dependently decreased MDA cell viability compared to the control group (25 µg/mL: 37.11%, *p* < 0.05; 50 µg/mL: 63.61%, *p* < 0.0001) (Figure 6). In addition, 25 and 50 µg/mL doses significantly decreased cell viability compared to the 12.5 µg/mL dose. SIM suspension and the unloaded cubosomes at all doses had no significant effect on MDA cell viability compared to the control. Furthermore, the 50 µg/mL dose significantly decreased cell viability compared to other doses of the SIM suspension and unloaded cubosomes. These results suggest that the SIM-cubosomal formula had a considerable effect on breast cancer cells, which could be attributed to the increased cellular uptake of SIM-loaded cubosomes compared to the SIM suspension [49,50].

## 4. Conclusions

SIM-loaded cubosomes were fabricated successfully using different glyceryl monooleate, Pluronic F127 (PF-127), and polyvinyl alcohol (PVA) ratios. This study demonstrated that PF-127 and PVA positively affected drug loading. Moreover, PF-127 positively affected the in vitro release rate of the different SIM cubosomes. The in vitro drug dissolution from the selected ODTs containing the cubosomal formula was high owing to the effect of the hydrophilic additive (mannitol) and the rapid disintegration of the tablets due to the effect of the superdisitegrant. In addition, the selected SIM-cubosomal formula was superior in cytotoxicity examinations over SIM suspensions and drug-free cubosomes which could be attributed to the increased cellular uptake of SIM-loaded cubosomes compared to the SIM suspension and drug-free cubosomes. Overall, this study provides novel insights into the oral administration of SIM via cubosomal formulae. However, more clinical pharmacodynamic and pharmacokinetic investigations are required to determine the clinical potential of the proposed cubosomes.

## Figures and Tables

**Table 1:** Composition and characterisation of different formulations of SIM-loaded cubosomes.

Formulation	Dispersed Phase (5% of Total Dispersion Weight)		Stabiliser	EE%	Particle Size (nm ± SD)	PDI (±SD)	Zeta Potential (mV ± SD)	% Drug Released
	% GMO	% Pluronic F127	% PVA					
F1	97.5	2.5	0	33.52 ± 1.55	188.3 ± 4.32	0.355 ± 0.03	−55.5 ± 0.17	40.78 ± 1.23
F2	95	5	0	51.20 ± 1.11	311.2 ± 10.13	0.600 ± 0.01	−51.0 ± 0.85	57.60 ± 2.45
F3	90	10	0	61.87 ± 1.46	316.6 ± 11.26	0.677 ± 0.02	−46.0 ± 0.61	81.71 ± 3.12
F4	95	2.5	2.5	40.05 ± 1.46	197.3 ± 4.98	0.369 ± 0.01	−57.9 ± 0. 11	29.42 ± 2.35
F5	92.5	5	2.5	57.41 ± 1.29	199.9 ± 3.88	0.352 ± 0.01	−54.6 ± 0. 15	43.57 ± 2.87
F6	87.5	10	2.5	70.39 ± 1.75	194.1 ± 3.01	0.292 ± 0.04	−47.6 ± 0. 15	60.71 ± 1.89
F7	92.5	2.5	5	49.71 ± 1.69	187.4 ± 3.56	0.349 ± 0.02	−56.3 ± 0. 55	25.88 ± 3.45
F8	90	5	5	65.09 ± 1.97	217.3 ± 5.23	0.401 ± 0.01	−53.5 ± 0. 25	37.80 ± 2.54
F9	85	10	5	80.80 ± 1.35	181.9 ± 3.64	0.352 ± 0.01	−47.7 ± 0. 78	51.45 ± 2.84

**Table 2:** Kinetic release data of different SIM-loaded cubosomes.

Formula	Zero-Order		First-Order		Higuchi Diffusion Model	
	R^2^	Slope	R^2^	Slope	R^2^	Slope
F1	0.9193	−9.814	0.9538	−0.0563	0.9863	−21.912
F2	0.8767	−13.814	0.9308	−0.0939	0.9737	−31.3812
F3	0.8751	−17.883	0.9864	−0.1723	0.9946	−41.0951
F4	0.9161	−7.29	0.9364	−0.0381	0.9717	−16.1828
F5	0.8683	−9.9166	0.9122	−0.0583	0.9801	−22.709
F6	0.8892	−13.549	0.9599	−0.0948	0.9950	−30.8936
F7	0.9330	−6.36	0.9494	−0.0322	0.9645	−13.9382
F8	0.9355	−8.96	0.9632	−0.0497	0.9859	−19.8257
F9	0.8984	−12.003	0.9496	−0.0764	0.9909	−27.1718

## Data Availability

The data are contained in the manuscript.
